# Autoclaving-Triggered Hydrogelation of Chitosan-Gluconic acid Conjugate Aqueous Solution for Wound Healing

**DOI:** 10.3390/gels9040280

**Published:** 2023-03-29

**Authors:** Yusuke Yamashita, Yoshihiro Ohzuno, Yoichi Saito, Yukio Fujiwara, Masahiro Yoshida, Takayuki Takei

**Affiliations:** 1 Department of Chemical Engineering, Graduate School of Science and Engineering, Kagoshima University, 1-21-40 Korimoto, Kagoshima 890-0065, Japanmyoshida@cen.kagoshima-u.ac.jp (M.Y.); 2 Laboratory of Bioengineering, Faculty of Advanced Science and Technology, Kumamoto University, 2-39-1 Kurokami, Chuo-ku, Kumamoto 860-8555, Japan; west-east@mech.kumamoto-u.ac.jp; 3 Department of Cell Pathology, Graduate School of Medical Sciences, Faculty of Life Sciences, Kumamoto University, 1-1-1 Honjo, Chuo-ku, Kumamoto 860-8556, Japan; fuji-y@kumamoto-u.ac.jp

**Keywords:** hydrogel, autoclave, sterilization, wound dressing, chitosan

## Abstract

Moist wound healing is known to heal wounds faster than dry wound healing. Hydrogel wound dressings are suitable for moist wound healing because of their hyperhydrous structure. Chitosan, a natural polymer, promotes wound healing by stimulating inflammatory cells and releasing bioactive compounds. Therefore, chitosan hydrogel has great potential as a wound dressing. In our previous study, physically crosslinked chitosan hydrogels were successfully prepared solely by freeze-thawing of chitosan-gluconic acid conjugate (CG) aqueous solution without using any toxic additives. Furthermore, the CG hydrogels could be sterilized by autoclaving (steam sterilization). In this study, we showed that autoclaving (121 °C, 20 min) of a CG aqueous solution simultaneously achieved gelation of the solution and sterilization of the hydrogel. Hydrogelation of CG aqueous solution by autoclaving is also physically crosslinking without any toxic additives. Further, we showed that the CG hydrogels retained favorable biological properties of the CG hydrogels prepared by freeze-thawing and subsequent autoclaving. These results indicated that CG hydrogels prepared by autoclaving were promising as wound dressings.

## 1. Introduction

Moist wound healing is more rapid and scars less than dry wound healing because it provides a moist and warm environment that is optimal for wound healing [1]. In a moist environment, a wound healed efficiently due to the presence of growth factors, cytokines, and immune cells such as polymorphonuclear leukocytes (PMN). Furthermore, macrophages facilitate the migration of epidermal basal cells and the progression of newborn blood vessels, resulting in efficient wound healing. Hydrogel wound dressings are suitable for moist wound healing because of their hyperhydrous structure. Typically, hydrogel wound dressings have been made from various synthetic and natural polymers [2,3].

Chitosan is a cationic natural polysaccharide composed of randomly repeating units of D-glucosamine and N-acetylglucosamine linked by β-1,4 glycosidic bonds. The polymer has been used in biomedical applications due to its excellent properties such as biocompatibility, non-cytotoxicity, good hemostatic property, and biodegradability [4,5,6,7]. Furthermore, chitosan stimulates the induction of polymorphonuclear neutrophils (PMN) and macrophages for phagocytosis [8]. They also promote the formation of granulation tissue and induce them into the proliferative phase [9]. These properties of chitosan show that chitosan hydrogel has great potential for wound dressing. However, typical chitosan hydrogels are acidic because chitosan is only soluble in acidic solvents because of its rigid crystal structure [10]. In addition, these hydrogels have been fabricated using toxic additives such as crosslinkers, polymerization initiators, and salts [11,12,13]. These properties limit the use of chitosan hydrogels as a wound dressing.

We previously reported that chitosan-gluconic acid conjugate (CG) dissolved in neutral aqueous solutions and CG hydrogels could be prepared by freeze-thawing the CG aqueous solution (cryogelation) without the use of additives [14]. The CG cryogels exhibited high biocompatibility and good biodegradability and were used for cartilage as well as bone tissue engineering [15,16]. Furthermore, the CG hydrogels accelerated wound healing because CG retained the favorable biological properties of chitosan [14,17]. The CG hydrogels were disinfected by using 70% ethanol aqueous solution before being applied to would [14,17]. Subsequently, we showed that CG hydrogels could be sterilized by autoclaving, which is steam sterilization at high temperature and high pressure, due to its high thermal resistance, and retained suitable biological properties of the pre-autoclaved CG hydrogels for wound healing [18]. Furthermore, autoclaving was shown to produce denser polymeric skeletons of the CG hydrogels, resulting in a higher resistance of the hydrogels to enzymatic degradation than the pre-autoclaved hydrogels [18]. The densification of a polymeric skeleton by autoclaving indicates the successful formation of CG hydrogels solely by autoclaving the CG aqueous solution.

The purpose of this study was to show that autoclaving of the CG aqueous solution simultaneously achieved gelation of the solution and sterilization of the hydrogel. Considering that our previous reports required two steps to prepare sterilized hydrogels (freeze-thawing for hydrogel formation and autoclaving for sterilization) [18], the single step in the present study is practical. Furthermore, physical crosslinking of a CG aqueous solution by autoclaving can also avoid the use of toxic additives. This is the first report of a simple autoclaving method for the preparation of chitosan hydrogels.

## 2. Results and Discussion

**Scheme 1:**
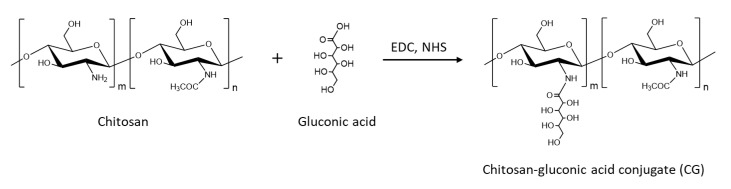
Schematic illustration of the synthesis of chitosan-gluconic acid conjugate.

In this study, CG was synthesized by incorporating gluconic acid into chitosan, as illustrated in Scheme 1, and three types of CG with different gluconic acid content were used (Table 1). Herein, the gluconic acid content is the number of gluconic acids modified per 100 glucosamine units of chitosan. CGs with an 8, 26, and 54 gluconic acid content were named CG8, CG26, and CG54, respectively. CG8 is chitosan with a little gluconic acid incorporated into it. CG54 incorporates as much gluconic acid as possible into chitosan. CG26 is of medium gluconic acid content. We considered that a comparison of these three types of CGs would adequately demonstrate the properties of CGs, so we planned to prepare hydrogels using these CGs and compared the performance of these hydrogels. In addition, we prepared CG hydrogels using two methods: CG hydrogels prepared by freeze-thawing CG aqueous solution and then sterilized by autoclaving were named FT-AC hydrogels, and CG hydrogels prepared only by autoclaving were named AC hydrogels.

### 2.1. Preparation of AC Hydrogels

In this study, the crystal structure of chitosan is important. Chitosan is obtained by partial deacetylation of chitin, which has repeating units of N-acetylglucosamine linked by β-1,4 glycosidic bonds [14]. Chitin is known to have a tight crystal structure consisting of numerous inter- and intramolecular hydrogen bonds [14]. Chitosan has a similar crystalline structure [14] and CG, a polymer of chitosan partly modified with gluconic acid, also retains a chitosan’s crystalline structure. Therefore, CG was expected to form hydrogels with the crystalline portion of CG as the crosslinking point.

**Scheme 2:**
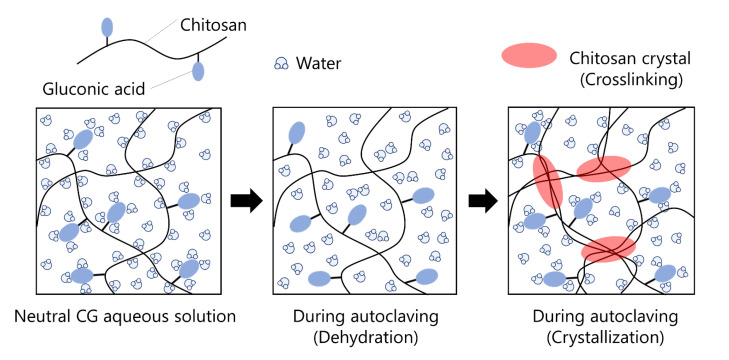
Mechanism of hydrogelation of chitosan-gluconic acid conjugate (CG) aqueous solution by autoclaving. When the neutral CG aqueous solution is heated by autoclaving, perturbation and dehydration occur, which makes crystallization superior to hydration, accelerating crystallization (crosslinking).

We hypothesized that the autoclaving-triggered hydrogelation of CG aqueous solutions occurs by the following mechanism. CG hardly dissolves in neutral water when it is added to neutral water directly due to the rigid crystalline structure of the chitosan main chain, although incorporated gluconic acid has improved its water solubility. On the other hand, CG dissolves in neutral water in the following procedure. CG is first dissolved in acidic water through electrostatic repulsion between protonated amino groups of unmodified D-glucosamine units. An aqueous solution of 0.1–1.0N NaOH is then added to the acidic CG solution gradually by stirring it to increase the pH to neutral. In the case of unmodified chitosan, the polymer precipitates at a pH of 6.0–6.5 due to the crystallization of the chitosan backbone caused by deprotonation of the amino groups. However, CG hardly precipitates even at neutral pH because the gluconic acid moiety in CG molecules sterically hinders the crystallization of the chitosan backbone. In the process, CGs with higher gluconic acid content were more soluble in water and therefore less likely to precipitate, which has been demonstrated in our previous report [14]. Considering that CG hardly dissolves in neutral water when it is added to neutral water directly, the neutral CG aqueous solutions are expected to be thermodynamically unstable. When the neutral CG aqueous solution is heated by autoclaving, perturbation occurs and dominates hydration, which leads to the dehydration of the polymer. Such perturbation and dehydration by heating a polymer solution were reported [19]. The dehydration of CG molecules would accelerate their crystallization (crosslinking) and follow hydrogel formation (Scheme 2). Thus, chitosan crystals would play an important role in the formation of CG hydrogel as a physical crosslinking point. Even after cooling the hydrogels to room temperature, the formed gel skeleton is expected to dissolve in the neutral water due to the crystallinity of CG because CG hardly dissolves in neutral water when it is added to neutral water directly, as described above.

**Figure 1:**
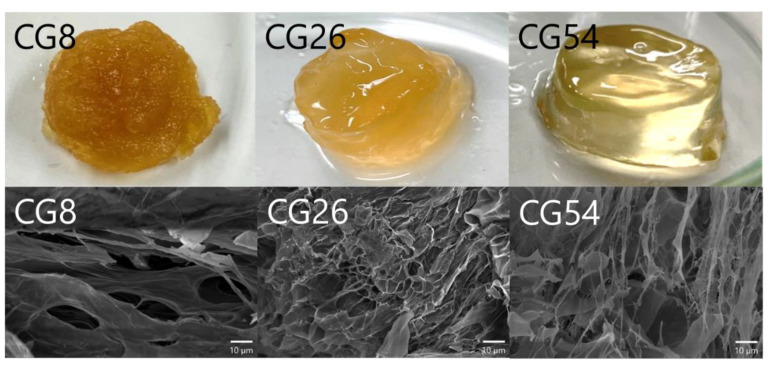
AC hydrogels prepared by autoclaving for 20 min (the hydrogels were prepared in a cylindrical vessel with a diameter of 15 mm) and scanning electron microscopic (SEM) images of their inner structure.

To prepare AC hydrogels, we first added solid CG powder to a dilute HCl aqueous solution (pH 4.0) and stirred it. For CG8 and CG26, the solid powder was completely dissolved in the acidic aqueous solution. On the other hand, for CG54, some of the powder remained undissolved. Subsequently, alkaline water was added to the acidic solution by stirring it to adjust the pH to 7.0. As expected, AC hydrogels were successfully prepared by autoclaving the neutral CG aqueous solution (Figure 1). CG8 aqueous solution formed a sponge-like hydrogel, while CG26 and CG54 aqueous solution formed smooth hydrogels through autoclaving. The structural difference can be seen more clearly in the SEM image. The CG8 AC hydrogel is shown to have a thick film gel skeleton with distinct large pores with a pore size of about 10–20 µm. The CG26 AC hydrogel has a gel skeleton that forms several small pores with a pore size of about 5–10 µm. The CG54 AC hydrogel has a very thin gel skeleton, forming a scabrous and fragile structure. AC hydrogel would form by crystallization of chitosan main chains. Therefore, we considered that the lower the gluconic acid content, the greater the influence of the chitosan main chain, which leads to the formation of a thicker hydrogel skeleton.

We investigated the formation of hydrogels by heating CG aqueous solutions at various temperatures and proved that heating was the trigger for the hydrogelation of CG aqueous solutions. Table 2 shows hydrogelation at various temperatures and times. At 100 °C, a hydrogel was formed in 20 min. However, no hydrogel was formed at temperatures lower than 100 °C. Furthermore, when heating was continued up to 120 min, the CG aqueous solution gelled even at 60 °C. Heating at 40 °C for several hours did not gel the solution, but after 3 days of heating, it did gel. To investigate gelation at further lower temperatures, the solution was incubated at room temperature, but it did not gel even after 7 days. The results indicate that the higher the heating temperature, the faster the gelation. In addition, considering that the gelation occurred even at a low temperature of 40 °C over a long period (3 days), it is conceivable that the neutral CG aqueous solution is in a metastable state and the crystal of chitosan as its stable state is formed by heating. The subsequent experiments were conducted using AC hydrogels, which were prepared by heating at 121 °C.

**Figure 2:**
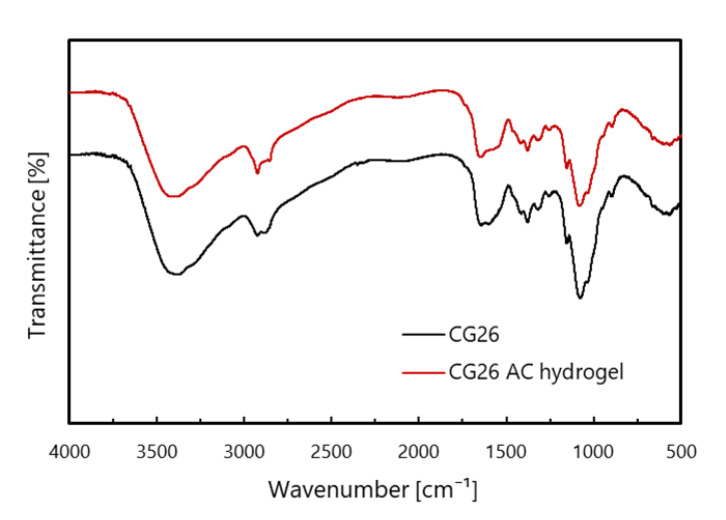
FT−IR spectra of CG26 before autoclaving and CG26 AC hydrogel.

We analyzed the AC hydrogels to clarify if any new covalent bonds were formed between the polymers by Fourier transform infrared (FT-IR) spectrometer. The FT-IR spectra of solid CG and AC hydrogel prepared by autoclaving is shown in Figure 2. Stretching vibration in the axial direction of the -NH and -OH groups was observed as the broad band around 3400 cm^−1^ [20]. The adsorption band at 1155 cm^−1^ is due to asymmetric stretching of the C-O-C bridge, and 1074 cm^−1^ and 1031 cm^−1^ indicate C-O stretching. These were attributed to the saccharide structure of CG [4,21]. The bands observed at 1654 cm^−1^ (amide I) and 1568 cm^−1^ (amide II) were characteristic of CG [4,22]. However, apparent new bands did not appear in AC hydrogel prepared by autoclaving. The results indicate that the hydrogelation of CG aqueous solution was attributed to physical crosslinking.

### 2.2. Mechanical Strength of AC Hydrogels

**Figure 3:**
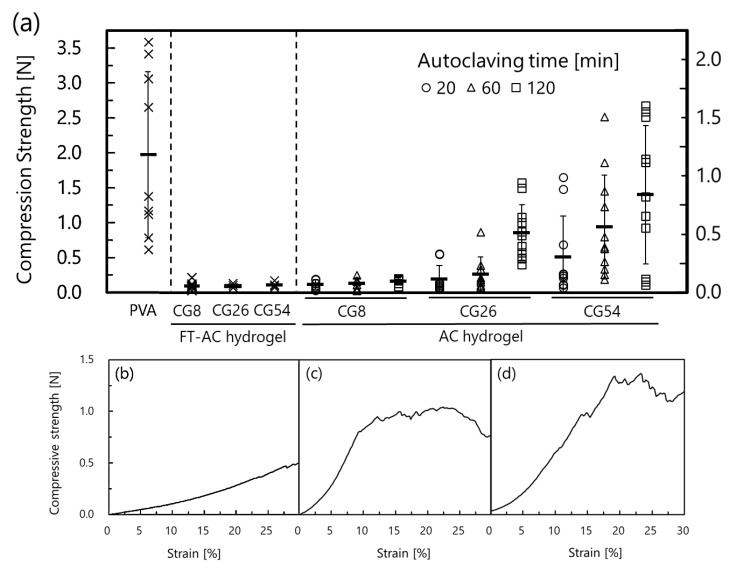
(**a**) The compressive strength at 10% strain of AC hydrogels prepared at different autoclaving times (20–120 min), FT-AC hydrogels, and poly(vinyl alcohol) (PVA) hydrogels (used as a major component of commercial wound dressing) (n = 6–12). The scale on the left vertical axis is for PVA hydrogel and the scale on the right vertical axis is for AC hydrogel and FT-AC hydrogel. Typical compressive strength vs strain curve of (**b**) CG8, (**c**) CG26, (**d**) CG54 AC hydrogels.

There have been no reports concerning chitosan hydrogels prepared by autoclaving. Therefore, knowledge concerning mechanical strength as a basic physical property of hydrogels is important. Herein, we performed compressive tests on AC hydrogels prepared and further investigated the effects of different gluconic acid content and autoclaving times on the compressive strength of the hydrogels. Figure 3a shows the compression strength of AC hydrogels prepared at different autoclaving times, FT-AC hydrogels, and poly (vinyl alcohol) (PVA) hydrogels. CG8 AC hydrogels exhibited lower compressive strength than the other AC hydrogels. The reason for this is that CG8 AC hydrogels have a denser gel skeleton than CG26 and 54 AC hydrogels, which allows water to escape and disperse the force when they are compressed (SEM images in Figure 1). The strength of CG8 AC hydrogels was independent of autoclaving time. CG26 AC hydrogels exhibited higher compressive strength than CG8 AC hydrogels. This is because the CG26 AC hydrogels retain water even under compression, as opposed to CG8 AC hydrogels. Furthermore, the longer autoclaving time, the higher the compressive strength of CG26 AC hydrogels, which means that the crystallization of CG was accelerated by autoclaving. CG54 AC hydrogels exhibit the highest compressive strength of the three. The strength increased with increasing autoclaving time, like CG26. However, the compressive strength of CG54 AC hydrogels was quite variable. This is due to the unstable solubility of CG54 which may have both soluble and insoluble portions in the polymer in the aqueous solution described above. AC and FT-AC hydrogels were inferior in strength to PVA hydrogels used as the main component of commercial wound dressings. Thus, in cases where high strength is required for wound dressing, it may need to be used in combination with other sufficiently durable materials. However, wound dressings need not only to have high mechanical strength but also to be flexible and deformable according to the shape and movement of the wound. Therefore, AC hydrogel, which is easily deformable, is expected to be suitable for effective treatment. Comparing the compressive strength of AC hydrogels and FT-AC hydrogels, the FT-AC hydrogels had similar compressive strength for all three types of CG, unlike AC hydrogels, which showed higher strength with higher gluconic acid content. FT-AC hydrogels are prepared by freeze-thawing, so crosslinking occurs gently, whereas AC hydrogels are prepared by heating, so crosslinking occurs intensively, and the difference is thought to cause the difference in strength. However, the reasons for the different strengths of these physically crosslinked CG hydrogels are not known in detail. Figure 3b–d shows typical compressive strength vs. strain curve. As previously mentioned, unlike CG26 and CG54 hydrogels, CG8 hydrogels are sponge-like and do not fracture when compressed, as shown in the figure.

### 2.3. Degradation of AC Hydrogels by Lysozyme

**Figure 4:**
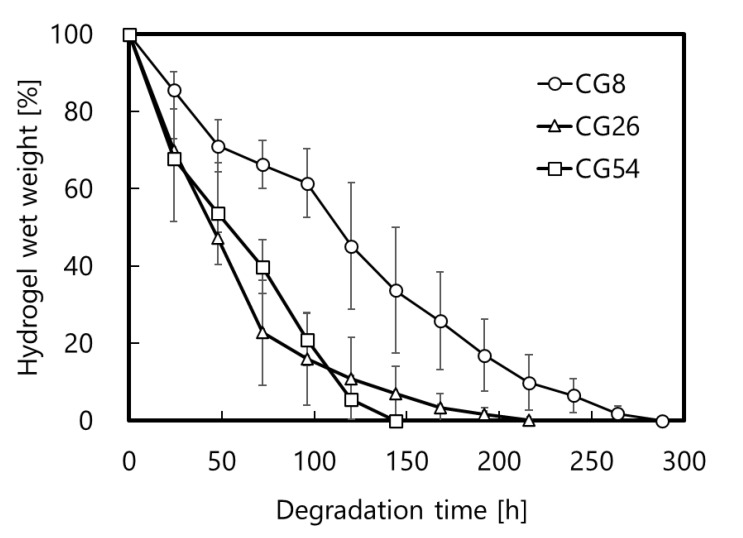
The degradation rate of CG8, CG26, and CG54 AC hydrogels by lysozyme (n = 4).

Chitosan is degraded by lysozyme in the body. Therefore, it is very important to investigate the degradability of AC hydrogels when using them as wound dressings. In this study, we investigated the effect of gluconic acid content on the degradability of AC hydrogels. CG54 AC hydrogels were completely degraded within 150 h (Figure 4). CG26 AC hydrogels were completely degraded over a longer period of more than 200 h. CG8 AC hydrogels took the longest time (290 h) to degrade completely. These results attributed to the difference in the total number of units of CG including the D-glucosamine unit, N-acetylglucosamine unit, and gluconic acid modified-D-glucosamine unit. Because of this, lysozyme hydrolyzes the β-1,4 glycosidic bonds between these units, which means that the larger the total number of units of CG, the longer the hydrogel takes time to be completely degraded. Considering the molecular weights of the D-glucosamine unit, the N-acetylglucosamine unit, and the gluconate-modified D-glucosamine unit are 161, 203, and 339, respectively, and the degree of deacetylation and the percentage of gluconic acid content, the average molecular weight of CG8, CG26, and CG54 are 180.6, 207.3, and 248.2, respectively. That is, the ratio of the total number of units present in the 2% (*w*/*v*) CG8, CG26, and CG54 AC hydrogels is 1.37:1.20:1.00, respectively. Based on this, the time it takes to degrade the CG AC hydrogels was longer with a decrease in gluconic acid content.

Considering that the compressive strength of CG54 AC hydrogels was quite variable due to the unstable solubility of CG54, either CG8 or CG26 AC hydrogel is suitable for wound treatment. Of the two AC hydrogels, CG26 AC hydrogel is less likely to dehydrate by means of compression, while CG8 AC hydrogel is easily dehydrated. Thus, CG26 AC hydrogel, which contains large amounts of water and is suitable for moist wound healing, was used for subsequent blood-clotting tests and wound treatments.

### 2.4. Blood-Clotting Test

**Figure 5:**
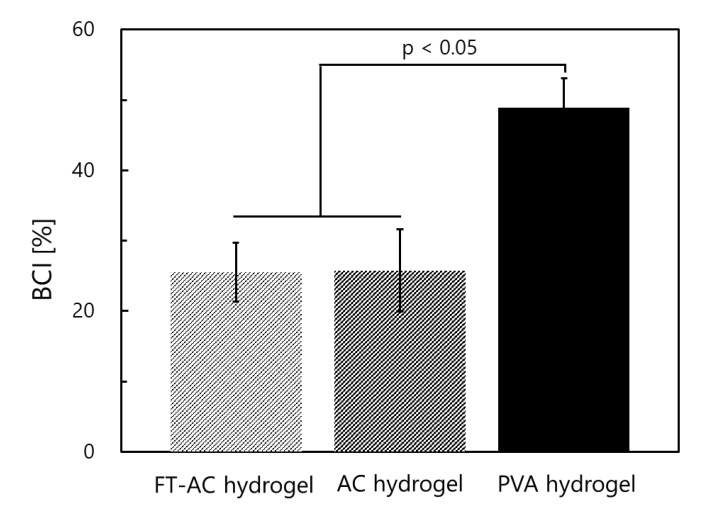
Hemostatic properties of CG26 AC hydrogels, CG26 FT-AC hydrogels, and PVA hydrogels prepared by freeze-thawing of PVA aqueous solution (n = 4).

It is important to investigate the hemostatic properties of AC hydrogels when using them as wound dressings. Chitosan, a positively charged polymer, electrostatically attracts negatively charged red blood cells and platelets, resulting in the formation of blood clots (hemostasis). Hemostatic properties of AC hydrogels were examined by means of a blood-clotting test. AC hydrogels (CG26) had a lower coagulation index than poly (vinyl alcohol) (PVA) hydrogels prepared by freeze-thawing of PVA aqueous solution (Figure 5). PVA was used because the polymer has been used as a commercial dressing material. These results show that AC hydrogels coagulate a large number of blood cells and have excellent hemostatic properties. Furthermore, there was no difference in the hemostatic properties between AC hydrogels and FT-AC hydrogel, showing that AC hydrogels retained favorable biological properties of FT-AC hydrogels.

### 2.5. Treatment of Wounds with AC Hydrogels

**Figure 6:**
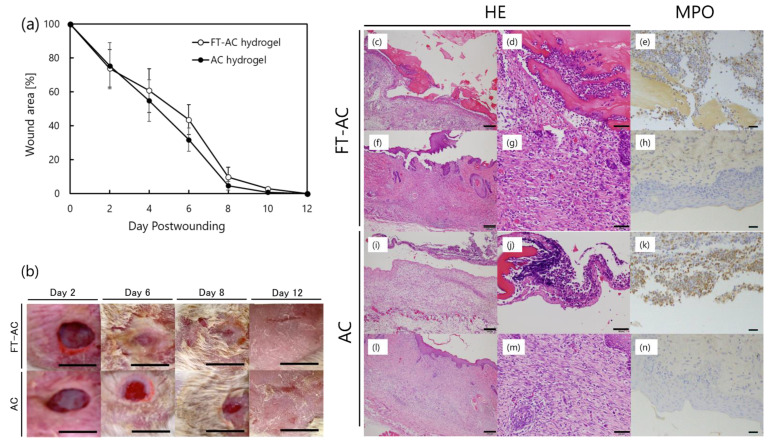
(**a**) Healing rate of wounds treated with CG26 AC and FT-AC hydrogels (day 0 and 2 n = 11, day 4 n = 10, day 6 n = 9, day 8 n = 8, day 10 n = 7, day 12 n = 3). (**b**) The macroscopic observation of wounds treated with FT-AC and AC hydrogel (Scale bars are 1 cm). (**c**–**n**) Hematoxylin- and eosin- (HE) (**c**,**d**,**f**,**g**,**i**,**j**,**l**,**m**) and immunohistochemically stained sections of myeloperoxidase (MPO) (**e**,**h**,**k**,**n**) of wound tissues treated with AC and FT-AC hydrogels of day 2 (**c**,**e**,**i**,**k**) and day 12 (**f**,**h**,**l**,**n**). (**d**,**g**,**j**,**m**) Magnified image of (**c**,**f**,**i**,**l**). Scale bars are 200 µm (**c**,**f**,**i**,**l**), 50 µm (**d**,**g**,**j**,**m**), and 100 µm (**e**,**h**,**k**,**n**), respectively.

Here, the effectiveness of CG26 AC hydrogels in healing full-thickness skin wounds was compared with CG26 FT-AC hydrogels. Our previous studies showed that (i) CG hydrogels prepared by freeze-thawing of CG aqueous solution and subsequent immersing in 70% ethanol for sterilization (designated as FT-ET hydrogels) accelerated wound healing more efficiently than commercially available hydrogel wound dressings [14] and (ii) FT-AC hydrogels had almost the same efficacy in repairing skin wounds as FT-ET hydrogels. Figure 6a shows the reduction in the wound area treated with AC or FT-AC hydrogels. At six days post-wounding, wounds treated with AC hydrogel and FT-AC hydrogel were 31.8 and 43.6% of initial wound area, respectively, and both healed more than 50% of the wound. Furthermore, at eight days post-wounding, AC hydrogel and FT-AC hydrogel mostly healed the wound to 4.6 and 9.7% of the initial wound area, respectively. Figure 6b also shows that the wound contracted as healing progressed, and it was observed that complete epithelialization was achieved at 12 days post-wounding. There was no difference between AC hydrogels and FT-AC hydrogels, both of which were completely healed in 12 days. Histological analysis showed a significant accumulation of many inflammatory cells in hematoxylin and eosin (HE) staining of both hydrogels at 2 days post-wounding (Figure 6c–n). Immunohistochemical staining of myeloperoxidase (MPO) as a neutrophil marker revealed that these inflammatory cells are neutrophils, which are capable of the secretion of chemical mediators that accelerate wound healing [8]. The histological reaction to both hydrogels, that numerous neutrophils accumulated, is typical for chitosan. At 12 days post-wounding, the hydrogel-treated defect area and infiltrated neutrophils were replaced by granulation tissue, and epithelialization of wound sites was completed in both hydrogels. These results show that AC hydrogels heal wounds as well as FT-AC hydrogels. A smaller number of processes in the present study (a single step) to prepare sterilized CG hydrogel than that in our previous study (2 steps) is practical for wound healing.

## 3. Conclusions

In conclusion, sterilized chitosan hydrogels were prepared by autoclaving chitosan-gluconic acid conjugate (CG) aqueous solution. The CG hydrogels showed that their mechanical strength could be controlled by changing autoclaving time. The lower the gluconic acid content of CG, the longer the hydrogel took to be completely degraded by lysolyme. The hydrogels coagulate a larger number of blood cells than PVA hydrogel, which is used commercially for wound dressing. For wound care, the hydrogels promoted the accumulation of inflammatory cells, especially neutrophils, and accelerated wound healing. The CG hydrogels prepared by autoclaving showed the same hemostatic properties and efficacy in wound care as the CG hydrogels prepared by freeze-thawing of CG aqueous solution and subsequent autoclaving. These results show that the CG hydrogels prepared by autoclaving are promising for wound dressing.

## 4. Materials and Methods

### 4.1. Materials

Chitosan with 83% of degree of deacetylation was purchased from KIMICA Corporation (Tokyo, Japan). Sodium gluconate, N-hydroxysuccinimide (NHS), Poly (vinyl alcohol) (PVA) (degree of saponification 96%, degree of polymerization 1000), and lysozyme from egg white were purchased from FUJIFILM Wako Pure Chemical Corporation (Osaka, Japan). 1-Ethyl-3-(3-dimethylaminopropyl) carbodiimide monohydrochloride (EDC) was purchased from Peptide Institute, Inc. (Osaka, Japan).

### 4.2. Synthesis of CG

Three types of CG with different gluconic acid contents were synthesized by incorporating gluconic acid into chitosan via 1-ethyl-3-(3-dimethylaminopropyl) carbodiimide hydrochloride and N-hydroxysulfosuccinimide-mediated condensation of the amino groups of chitosan and carboxy groups of gluconic acid in an aqueous solution according to our previous report [14]. Chitosan was dissolved in 2-morpholinoethanesulfonic acid (MES) aqueous solution (pH 4.0). EDC, NHS, and sodium gluconate were dissolved in the chitosan solution. The molar ratios of sodium gluconate, EDC, and NHS to the amino group of chitosan used for the synthesis and the concentration of chitosan in the solution are shown in Table 1. The mixture was gently shaken at 30 °C for 24 h. The pH was then adjusted to 8.0 by adding 6 M NaOH, followed by adding the excess amount of 99% (*v*/*v*) ethanol. The precipitate was collected by filtration and dialyzed in deionized water using a dialysis membrane (MWCO: 14,000). Dialysis was continued until the conductivity of the dialyzed water was equal to that of deionized water. The purified CG was lyophilized to obtain CG sponge, which was then ground to CG powder. The gluconic acid content of the CG was determined by colloidal titration. The content was defined as the number of gluconic acids modified per 100 glucosamine units of chitosan. CG with 8, 26, and 54 of gluconic acid content were named CG8, CG26, and CG54, respectively. CG with gluconic acid content higher than 54% was not synthesized because the solution gelled during synthesis and was therefore difficult to purify.

### 4.3. Preparation of AC Hydrogels

CG was dissolved in a dilute HCl aqueous solution at a concentration of 2% (*w*/*v*) (pH 4.0). The pH was adjusted to 7.0 by adding 0.1–1.0M NaOH aqueous solution gradually. The CG solution was poured into a glass vial and autoclaved (121 °C, 20–120 min) or heated in a water bath (40–100 °C, 20 min—7 d). Then, it was left at room temperature to cool down.

SEM (S-3000N, Hitachi, Ltd., Tokyo, Japan) was used for observation of inner structure of AC hydrogels, which was instantaneously frozen in liquid nitrogen and then dried in vacuum. CG powder and freeze-dried AC hydrogels were ground into a powder, mixed with KBr, and molded into a disk shape by compressing. FT-IR spectra of the CG samples were recorded using an FT-IR spectrometer (Spectrum One, PerkinElmer, Inc., Waltham, MA, USA).

### 4.4. Compression Test

CG aqueous solutions were prepared at a concentration of 5% (*w*/*v*) and autoclaved (20–120 min) in a 15 mm diameter glass vial to prepare AC hydrogels. PVA aqueous solutions were prepared by autoclaving (121 °C, 30 min) the 15% (*w*/*w*) mixture of PVA and distilled water. The PVA aqueous solution was poured into a 15 mm diameter glass vial and cooled slowly at room temperature. The solution was frozen at 30 °C and then thawed at room temperature. The freeze-thawing process was performed again. These hydrogels with 15 mm diameter and 10 mm height in vials were compressed using a universal testing machine (RTC-1210A, ORIENTEC Co., Ltd., Tokyo, Japan). Compression strength of the hydrogels at 10% strain was used for comparison.

### 4.5. Degradation of AC Hydrogels by Lysozyme

AC hydrogels were prepared by autoclaving 2% (*w*/*v*) CG aqueous solutions (15 mm in diameter and 10 mm in height). The hydrogels were immersed in phosphate-buffered saline without calcium and magnesium ions (pH 7.4) containing 0.3% lysozyme and gently shaken at 37 °C. The weight of the hydrogels was determined at predetermined intervals.

### 4.6. Blood-Clotting Test

PVA was dissolved in heated deionized water at 5% (*w*/*v*) concentration. The PVA aqueous solution was poured into a cylindrical container with a diameter of 10 mm and frozen at −30 °C for 6 h and then left at room temperature to thaw. CG hydrogels were prepared by freeze-thawing or autoclaving (121 °C, 20 min, AC hydrogel) of 2% (*w*/*v*) CG26 aqueous solution poured into a cylindrical container with a diameter of 10 mm. CG hydrogels prepared by freeze-thawing were autoclaved (121 °C, 20 min) to prepare FT-AC hydrogels. FT-AC, AC, and PVA solid sponges were prepared by freezing instantaneously in liquid nitrogen and drying in vacuum. Blood was sampled from the abdominal aorta of Wister rats (male, 6 weeks old) under gas anesthesia with isoflurane. Whole blood was prepared by mixing the blood and 109 mM sodium citrate aqueous solution at a ratio of 9:1 (*v*/*v*) at 37 °C to prevent coagulation. Notably, 50 μL of the whole blood was dropped onto the solid sponges to absorb, followed by adding 5 μL of 0.2 M calcium chloride aqueous solution and then incubated at 37 °C for 5 min. The sponges were immersed in 6.25 mL of deionized water and incubated at 37 °C with shaking at 30 rpm for 10 min. Red blood cells that were not captured in blood clots were hemolyzed into water. By measuring the absorbance at 540 nm, the concentration of hemoglobin released from hemolyzed red blood cells into water was determined. As a reference, the absorbance of deionized water directly added to the same volume of whole blood was used. The blood-clotting index (BCI) was determined by the following equation:

BCI [%] = (absorbance of sample/absorbance of reference) × 100(1)

All animal experiments were performed according to the recommendations of Kagoshima University’s “Guide for the Care and Use of Laboratory Animals.”

### 4.7. Treatment of Wound with AC Hydrogels

2% (*w*/*v*) FT-AC (control) and AC hydrogels were prepared using CG26 in a cylindrical container with a diameter of 23 mm. Autoclaving condition was 121 °C and 20 min. Eleven male Wister rats (6 weeks old) were used in the experiment. Dorsal skin of Wister rats was shaved under anesthesia with mixed anesthetic agents (medetomidine hydrochloride, midazolam, and butorphanol tartrate) and treated with 70% ethanol to prevent infection. Two circular full-thickness skin wounds of 10 mm in diameter were created in dorsal skin of each rat using scissors. Each wound was covered with an AC hydrogel or an FT-AC hydrogel. The wounds were further covered with an adhesive waterproof film (BFR, Nichiban, Tokyo, Japan) as a second dressing. These dressings were securely fixed with an elastic adhesive bandage (SKINERGATE GACHITT, Nichiban, Tokyo, Japan). The shapes of wound were traced over a transparent film every other day. The wound areas were determined from the traces of wound using image analysis software (Image J). The rats were sacrificed by overdosing anesthesia with isoflurane one by one, and the entire wound, including adjacent normal skin, was excised for histological analysis.

### 4.8. Histological Analysis

Formalin-fixed (10 wt% neutral buffered) (Wako) paraffin-embedded specimens of the rat wound tissues were cut into 3 μm serial sections for hematoxylin and eosin staining or immunostaining of MPO. For antigen retrieval, the sections were immersed in EDTA (pH8) and heated in a microwave. The sections were incubated with rabbit polyclonal anti-MPO antibodies (A0398; Dako). Then, the sections were incubated with HRP-labeled goat anti-rabbit secondary antibodies (Nichirei Bioscience Inc., Tokyo, Japan). Immunoreactivity was visualized using 3–3 Diaminobenzidine Dab Substrate Kits (Nichirei Bioscience Inc., Tokyo, Japan).

## Figures and Tables

**Table 1:** Feeding molar ratios of sodium gluconate, EDC, and NHS to glucosamine units to synthesize the three types of CG and the gluconic acid content of the CG.

Name	Glucosamine Units	Sodium Gluconates	EDC	NHS	Concentration of Chitosan [% (*w*/*v*)]	Gluconic Acid Content /100 Glucosamine Units
CG8	1	0.25	0.25	0.125	2	8.0
CG26	1	0.5	0.5	0.25	2	26.3
CG54	1	2	2	1	1	54.0

**Table 2:** Hydrogelation of CG26 aqueous solution by heating at various temperatures (room temperature—121°C) and times (20 min—7 d).

		Temperature [°C]					
		121	100	80	60	40	Room Temp.
Time	20 min	+	+	−	−	−	−
	120 min	+	+	+	+	−	−
	3 d	ND	ND	ND	ND	+	−
	7 d	ND	ND	ND	ND	ND	−

+: Formed a hydrogel; −: Remained liquid; ND: No data.

## Data Availability

Data presented in this study are available on request from the corresponding authors.
